# Molecular identification of immature stages of medically important fly species, Puducherry, South India: a preliminary study

**DOI:** 10.3389/finsc.2025.1551807

**Published:** 2025-05-15

**Authors:** Sudha Bhuvaneshwaran, Visa Shalini Padmanaban, Ranjana Devi Radja, Gayathri Anandan, Shakila Venkatesan, Janani Semalaiyappan, Ashwani Kumar, Vijesh Sreedhar Kuttiatt

**Affiliations:** ICMR - Vector Control Research Centre, Puducherry, India

**Keywords:** maggots, mitochondrial cytochrome oxidase subunit I (COI), DNA barcoding, haplotype analysis, molecular methods

## Abstract

Flies and maggots are of medical importance, and it is often necessary to identify them at species level. Conventionally, this is carried out based on morphological features using taxonomic keys. However, identification of maggots based on morphology is difficult and required entomological expertise is often lacking in clinical settings. Molecular methods can be an alternative to morphology-based identification and find special application when only tiny pieces of specimens are available especially in cases of human myiasis. In this preliminary study, we explored the utility of mitochondrial COI gene based molecular method, for identifying immature stages of certain medically important flies captured from the field in Puducherry, India. Maggots were captured from different locations in Puducherry using rotten fish and kitchen waste as baits and a 700 bp segment of the COI gene was amplified and genetic relationship was assessed by performing haplotype network analysis. High quality sequences were available for 11 specimens and were subjected to BLAST analysis to identify matches from the database for identification of the species. The identified maggots belonged to *Sarcophaga peregrina* (Robineau-Desvoidy, 1830), *Hemipyrellia ligurriens* (Wiedemann, 1830) and *Chrysomya megacephala* (Fabricius, 1794). This study generated representative molecular sequence data for two less studied fly species of medical importance, *S. peregrina* and *H. ligurriens* from South India. In future, there is a need for further detailed molecular studies on flies in the diverse epidemiological and geographic settings in India with a view to identify cryptic species and new haplotypes.

## Introduction

Flies are of great medical significance as some adult flies transmit several deadly diseases to animals and humans as mechanical and obligatory vectors (1). Maggots of flesh flies (Sarcophagidae) and blue bottle flies (Calliphoridae) found on dead bodies are of forensic importance in estimation of post-mortem interval (PMI) (2). Myiasis is a condition where maggots infest live vertebrates (including humans) (1). In obligatory myiasis, the parasite is dependent on the host whereas it is not so in facultative myiasis (1). Many different species of flies are known to be associated with myiasis in human beings (1, 3). Though myiasis is harmful, in some instances, maggots are of medical benefit as well. For example, maggots are utilised for debridement therapy where sterile larvae of certain flies like green bottle fly (*Lucilia sericata (*Meigen, 1826*)*), are applied to clean up leg ulcers, pressure sores and infected surgical wounds (4).

Species level identification of flies and maggots is often necessary and conventionally, carried out based on morphological features by trained taxonomists. Identification is challenging when specimen lacks morphological characters due to damage happened while collection, transportation or by inappropriate preservation. Also, unlike adult flies, species identification of maggots is difficult as the larvae of different flies, especially the first instar stage appears similar, and the required entomological expertise and qualified professionals are often lacking in many settings. We recently came across a case of extensive myiasis of the leg in a patient with filariasis-related elephantiasis where we could not identify the larva because of the practical difficulties in collection of the maggots on his first visit to the Filariasis Management Clinic at our centre, and later he did not turn up for follow up (5). This is not an isolated scenario and in many published reports on myiasis worldwide, larval species identification is missing, often due to lack of entomological expertise in clinical settings. Molecular techniques offer the best alternative in this scenario (6, 7).

Molecular barcoding-based identification has been applied for animals including insects. However, molecular methods for identification of flies or maggots are not widely applied in the Indian setting (8–11). Haplotype network and phylogenetic analysis are especially useful for exploring the genetic variability in the regional and global context. However, when considering medically important flies, in the public domain, there are only limited number of gene sequences of flies like *C. megacephala*, *H. ligurriens* and *S. peregrina* from India especially from the southern region. This prodded us to carry out a preliminary study in Puducherry, applying cytochrome oxidase subunit I (COI) gene based molecular analysis for identification of field captured immature stages of certain medically important flies. We had two broad objectives, namely, capacity building for molecular barcoding of flies in our centre and generation of representative gene sequences of these flies from this part of the country. Additionally, in future, we want to employ this molecular technique routinely for identification of maggots when cases with myiasis present to our Filariasis Management Clinic.

## Materials and methods

**Figure 1:**
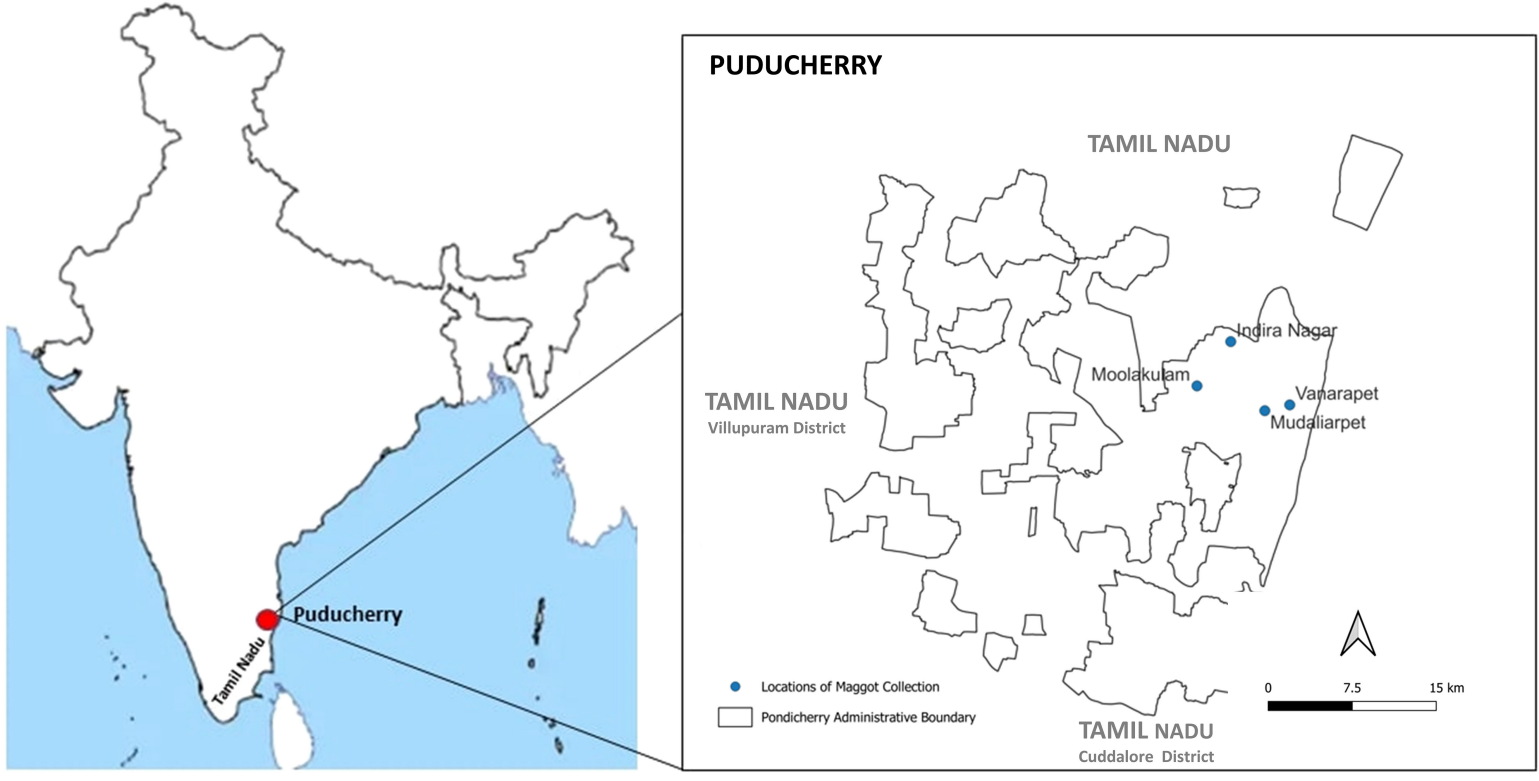
Sites of maggot collection in Puducherry.

Puducherry, is a union territory on the eastern coast in South India. We collected maggots from four sites of Puducherry, namely Vanarapet, Mudaliarpet, Moolakulam and Indira Nagar (**Figure 1**) using rotten fish and kitchen waste as baits. We also attempted to rear them in laboratory condition, but we were successful in only a few cases (12). Morphological evaluation was performed using the published taxonomic keys (13). Maggots and flies were processed individually for isolation of the mitochondrial DNA and a partial 710 bp segment of the COI gene was amplified using a set of universal primers as previously described (Forward: 5’-GGT CAA CAA ATC ATA AAG ATA TTG G-3’ Reverse: 5’-TAA ACT TCA GGG TGA CCA AAA AAT CA-3’. PCR conditions as below were employed - 95°C -5 min, 95°C- 1 min, 55°C - 1.30 min, 72°C- 1 min, 72°C 10 min and 4°C – infinity (14, 15). The amplicons were subjected to bidirectional cycle sequencing reaction using BigDye™ Terminator v3.1 cycle sequencing kit and capillary electrophoresis was performed on a 3130XL Genetic Analyser (Applied Biosystems, USA). The forward and reverse sequences were edited and trimmed using BioEdit version 7.7.1 software and consensus sequences generated. High quality sequences were available for 11 specimens and were subjected to BLAST analysis to identify matches from the database for identification of the species.

For comparative analysis, COI gene sequences of each of these species available in GenBank from India and other countries were downloaded. The multiple alignment was carried out using the ClustalW Multiple alignment program. MEGA 11 was used for molecular evolutionary analysis using the Neighbor-Joining (NJ) approach (16), using the Kimura 2-parameter (K2P) method (16, 17), pairwise nucleotide sequence divergences and mean evolutionary distances were calculated. Haplotype analysis for each species were carried out with DNASP 6.12 and the haplotype networks were visualized with the PopART (Population Analysis with Reticulate Trees) network analysis software using the median joining network (18).

## Results

On BLAST analysis, two sequences were found belonging to *Chrysomya megacephala* (VCRC IM3, IM5), three were identified as *Hemipyrellia ligurriens* (VCRC IFOG2A, IFOG3AF, IIF4), and six were *Sarcophaga peregrina* (IFOG4A, IRF3, IIRF4, F1IRM1F, IIRM2, IFOGM1). The COI nucleotide sequences generated from the maggots and the corresponding emerged adults were found to be of >99% matching. The maggots collected from Vanarapet belonged to *Sarcophaga peregrina* whereas the ones from Indira Nagar were *Hemipyrellia ligurriens* and the ones from Mudaliarpet and Moolakulam were *Chrysomya megacephala*.

**Figure 2:**
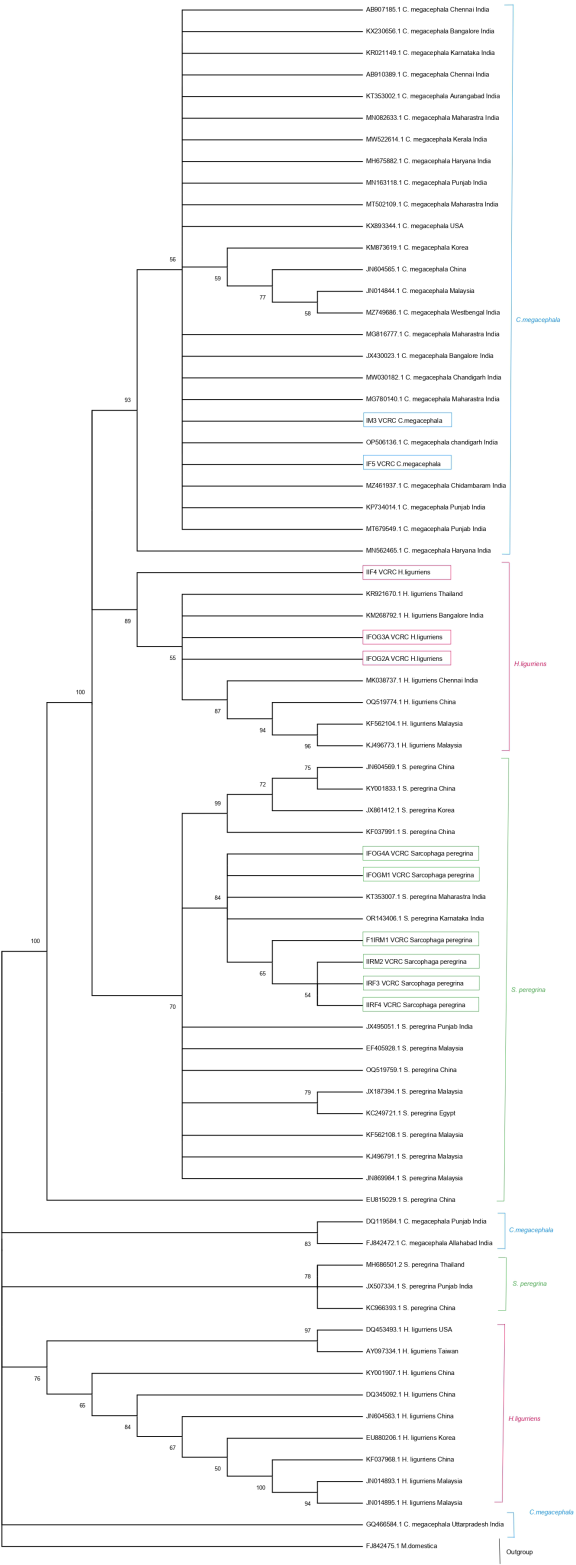
Neighbor-joining tree of 11 mitochondrial COI gene sequences (highlighted in box) generated from this study and the sequences reported in GenBank.

A total of 11 sequences generated from maggots were analyzed and compared with publicly available sequences from GenBank, including 31 COI sequences of *C. megacephala*, 16 of *H. ligurriens* and 19 sequences of *S. peregrina*. Sequences shorter than 500 base pairs were excluded, resulting in 27 sequences of *C. megacephala*, 15 of *H. ligurriens*, and 18 of *S. peregrina* being selected for further analysis alongside the newly generated sequences. Newly generated sequences have been submitted to GenBank (PP593642 to PP593652). We carried out haplotype analysis separately for the three species with sequences from India and other countries which revealed that there were 9, 6 and 10 haplotypes present in *C. megacephala, H. ligurriens and S. peregrina* respectively. It is to be noted that *C*. *megacephala* and *H. ligurriens* have less genetic divergence than *S. peregrina* (**Tables 1A**, **B**). Blast comparisons were made for all species, and the first records are reported in **Table 2**. This showed that the analyzed maggot sequences had a similarity of 98–100% to sequences already available in the public database. The results indicate that the maggots in this study belonged to three different species and are confirmed by neighbour-joining analysis as well (**Figure 2**).

**Table 1A:** Diversity indices calculated for all each species.

Species	*C. megacephala*	*H*. *ligurriens*	*S. peregrina*
Sample size	29	18	24
No. haplotypes (Nh)	9	6	10
No. segregating sites (S)	164	30	91
Haplotype diversity h	0.48	0.49	0.89
Variance of haplotype diversity	0.013	0.020	0.001
Standard deviation of haplotype diversity	0.115	0.142	0.036
Nucleotide diversity π (per site)	0.097	0.013	0.298

**Table 1B:** Genetic distance within and among species calculated for each species.

Species	*C. megacephala*	*H*. *ligurriens*	*S. peregrina*
*C. megacephala*	**0.19**	**-**	**-**
*H. ligurriens*	0.232	**0.02**	**-**
*S. peregrina*	0.626	0.487	**0.55**

Values highlighted in bold indicate genetic distance within a species.

**Table 2:** BLAST analysis of COI gene sequences of maggots.

Species	Accession No	Query Cover	E value	Similarity (%)	Matched Organism
IM3_VCRC *C.megacephala*	MZ461937.1	100%	0	99.38	*C. megacephala*
	OK560150.1	100%	0	99.22	*C. megacephala*
	KY020769.1	100%	0	99.06	*C. megacephala*
	MG557664.1	100%	0	99.06	*C. megacephala*
	MZ769396.1	100%	0	99.06	*C. megacephala*
	MF322594.1	100%	0	99.06	*C. megacephala*
	KT894991.1	100%	0	99.06	*C. megacephala*
IF5_VCRC *C.megacephala*	MZ461937.1	99.00%	0	100	*C. megacephala*
	FJ614817.1	100.00%		99.5	*C. megacephala*
IFOG2A_VCRC *H.ligurriens*	KR921676.1	100.00%	0	99.85	*H. ligurriens*
	KY001908.1	100.00%	0	99.85	*H. ligurriens*
	KJ496774.1	100.00%	0	99.85	*H. ligurriens*
	KF037968.1	100.00%	0	99.85	*H. ligurriens*
	EU880206.1	100.00%	0	99.85	*H. ligurriens*
	OQ519774.1	100.00%	0	99.85	*H. ligurriens*
	JN604563.1	100.00%	0	99.85	*H. ligurriens*
IFOG3AF_VCRC *H.ligurriens*	KY001908.1	100.00%	0	99.85	*H. ligurriens*
	KJ496774.1	100.00%	0	99.85	*H. ligurriens*
	KF037968.1	100.00%	0	99.85	*H. ligurriens*
	EU880206.1	100.00%	0	99.85	*H. ligurriens*
	OQ519774.1	100.00%	0	99.85	*H. ligurriens*
	JN604563.1	100.00%	0	99.85	*H. ligurriens*
	KR921676.1	100.00%	0	99.85	*H. ligurriens*
IIF4_VCRC *H.ligurriens*	MN831480.1	98%	0	99.84	*H. ligurriens*
	KR921676.1	100%	0	99.38	*H. ligurriens*
	KY001908.1	100%	0	99.38	*H. ligurriens*
	KJ496774.1	100%	0	99.38	*H. ligurriens*
	KY001903.1	100%	0	99.38	*H. ligurriens*
	KF037968.1	100%	0	99.38	*H. ligurriens*
	EU880206.1	100%	0	99.38	*H. ligurriens*
	OQ519774.1	100%	0	99.38	*H. ligurriens*
	JN604563.1	100%	0	99.38	*H. ligurriens*
IFOG4A_VCRC *S.peregrina*	PP784528.1	99.00%	0	100	*S. peregrina*
	KT353007.1	97.00%	0	100	*S. peregrina*
	EF405928.1	100.00%	0	98.66	*S. peregrina*
IRF3_VCRC *S.peregrina*	PP784528.1	100.00%	0	99.69	*S. peregrina*
	KT353007.1	97.00%	0	99.68	*S. peregrina*
	KC855283.1	100.00%	0	98.47	*S. peregrina*
	KJ496794.1	100.00%	0	98.47	*S. peregrina*
IIRF4_VCRC *S.peregrina*	PP784528.1	97	0	99.55	*S. peregrina*
	KJ496794.1	99	0	98.41	*S. peregrina*
	KC855283.1	99	0	98.4	*S. peregrina*
	EF405928.1	99	0	98.26	*S. peregrina*
F1IRM1F_VCRC *S.peregrina*	PP784528.1	100	0	99.53	*S. peregrina*
	KT353007.1	97	0	99.52	*S. peregrina*
	KC855283.1	100	0	98.27	*S. peregrina*
	KJ496794.1	100	0	98.27	*S. peregrina*
	EF405928.1	100	0	98.12	*S. peregrina*
	PP784528.1	100	0	99.4	*S. peregrina*
IIRM2_VCRC *S.peregrina*	KT353007.1	97	0	99.38	*S. peregrina*
	KC855283.1	100	0	98.2	*S. peregrina*
	KJ496794.1	100	0	98.2	*S. peregrina*
	EF405928.1	100	0	98.05	*S. peregrina*
IFOGM1_VCRC *S.peregrina*	PP784528.1	98	0	99.85	*S. peregrina*
	KT353007.1	97	0	99.85	*S. peregrina*
	EF405928.1	100	0	98.53	*S. peregrina*
	KC855283.1	100	0	98.38	*S. peregrina*
	KJ496791.1	100	0	98.38	*S. peregrina*

**Figure 3:**
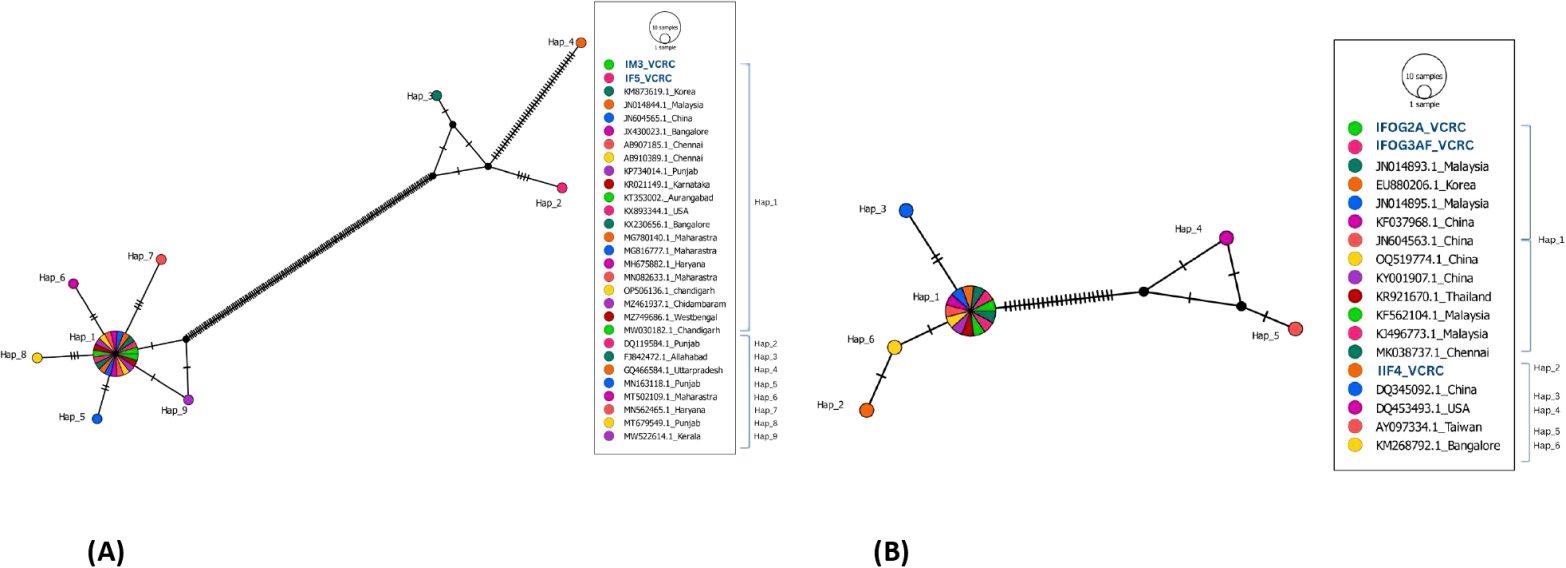
**(A)** Haplotype network of 27 COI sequences from *C. megacephala* belonging to India and other countries compared with 2 COI sequences (highlighted in blue) of *C. megacephala* of this study. **(B)** Haplotype network of 15 COI sequences from *H. ligurriens* that are from India and other countries compared with 3 COI sequences (highlighted in blue) of this study.

Haplotype network analysis demonstrates the evolutionary relationships between the haplotypes that have been identified. Newly generated two *C. megacephala* sequences (IM3_VCRC, IF5_VCRC) exhibited a strong clustering pattern and close association with every sequence analysed (**Figure 3A**). In BLAST analysis, these two sequences of *C. megacephala* showed a high similarity (over 97%) with 24 specific sequences retrieved from GenBank. However, GenBank sequences from Punjab, Allahabad and Uttar Pradesh (DQ119584.1, FJ842472.1, and GQ466584.1) exhibited a noticeable divergence of over 3%. These three sequences showed the highest nucleotide diversity, leading to the emergence of three haplotypes (Hap_2, Hap_3, and Hap_4). Furthermore, they were segregated into different haplogroups (**Figure 3A**). The newly generated sequences exhibited 100% similarity to the GenBank sequences classified under haplotype Hap_1. With minimal nucleotide differences, the remaining haplotypes were separated into distinct haplogroups. These nucleotide differences are shown in **Supplementary Additional File 1**.

In case of *H. ligurriens*, the three newly generated sequences from Puducherry showed over 99% similarity with 13 specific sequences retrieved from GenBank. However, sequences from Taiwan and the USA (AY097334.1, DQ453493.1), which were also obtained from GenBank, showed lower similarity (92–94%), placing them in a different haplogroup (Hap_4 and Hap_5). They showed the highest nucleotide diversity among the identified haplotypes (**Supplementary Additional File 1**). Hap_1 shows a close relationship between IIF4_VCRC and a Bangalore sequence. With the presence of two single nucleotide polymorphism, the sequence IIF4_VCRC was categorized under the haplotype Hap_2 in the network (**Figure 3B**).

**Figure 4:**
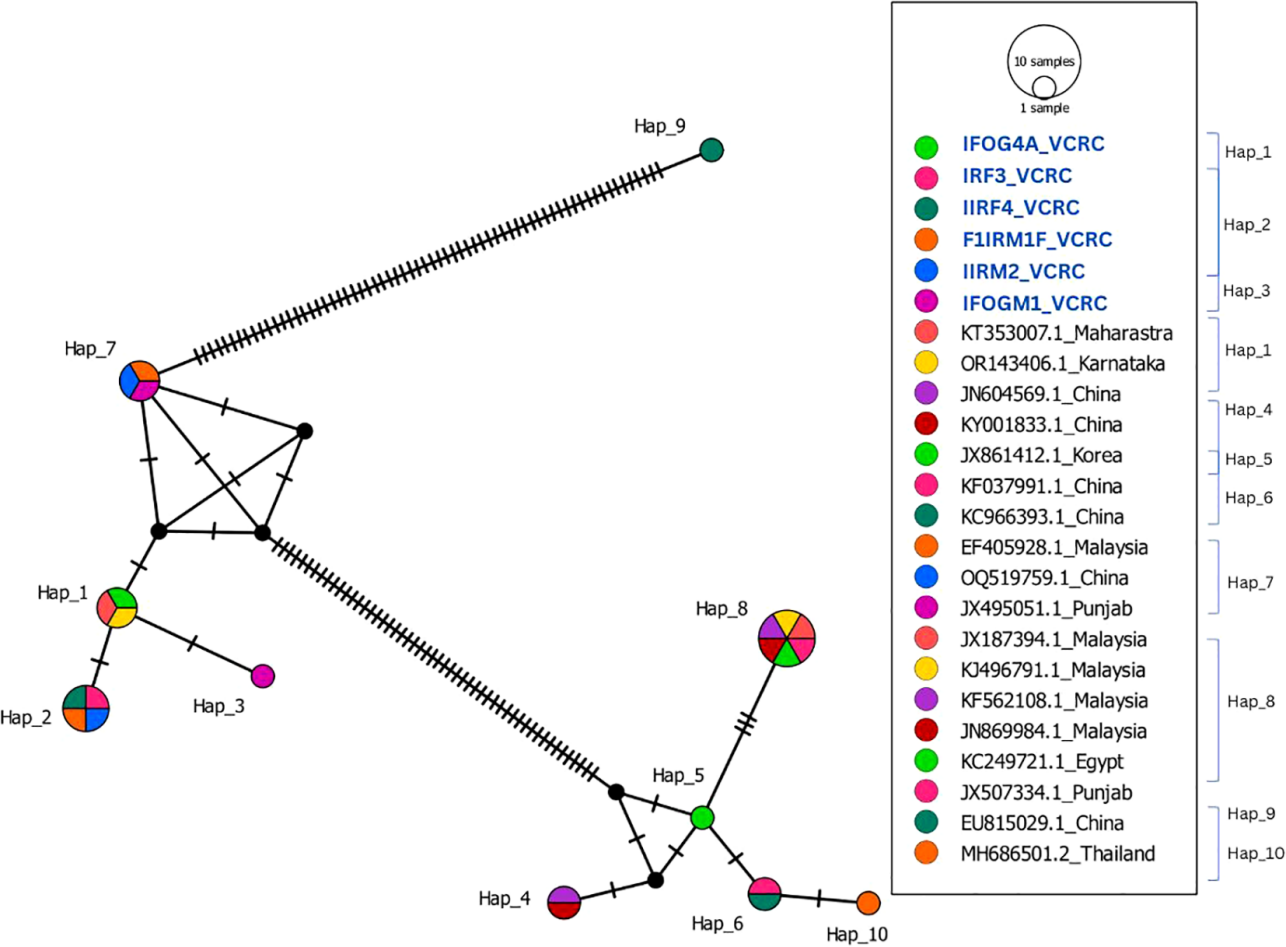
Haplotype network of 18 COI sequences from *S. peregrina* that are from India and other countries compared with 6 COI sequences (highlighted in blue) of *S. peregrina* of this study.

The six *S. peregrina* sequences from this study showed 97% similarity with 15 selected sequences from GenBank. However, other GenBank sequences from Punjab, Thailand, and China (JX507334.1, MH686501.2, EU815029, and KC966393.1) exhibited more than 3% divergence, indicating genetic differentiation. In addition to being categorised in distinct haplogroups, which comprise haplotypes Hap_6, Hap_9, and Hap_10, these sequences are more divergent from the newly generated sequences in this study. In Hap_1, Hap_2, and Hap_3, a cluster of six sequences from Puducherry, Maharashtra, and Karnataka (KT353007.1 and OR143406.1) was observed (**Figure 4**). The sequence IFOG4A_VCRC was 100% similar to the two GenBank sequences (KT353007.1_Maharastra, OR143406.1_Karnataka), showing that they belong to Hap_1. The sequences IRF3_VCRC, IIRF4_VCRC, F1IRM1_VCRC, and IIRM2_VCRC grouped into a single haplotype (Hap_2), whereas IFOGM1_VCRC formed a distinct haplotype (Hap_3). These two haplotypes were found to differ by a SNP (**Supplementary Additional File 1**). Despite this genetic distinction, the haplotype network analysis indicates that Hap_2 and Hap_3 remain closely related, suggesting recent divergence within the population (**Figure 4**). The neighbour-joining analysis revealed the presence of three separate clades, which correspond to the three species identified in this study (**Figure 2**).

## Discussion

In the current study, we employed mitochondrial COI gene based molecular method for identification of immature stages of flies captured from the field and found it a useful alternative to morphology-based identification. We generated partial COI gene sequences from three different species of medically important flies namely *C. megacephala*, *H. ligurriens* and *S. Peregrina* and these are deposited in GenBank. Haplotype network analysis revealed the genetic diversity and indicated the presence of multiple haplotypes. Although no direct evidence of cryptic speciation for these species was reported previously, studies have shown that it exhibits a wide geographical distribution and significant genetic divergence among populations. The observed genetic divergence in the networks in the present work suggests that, at least for *S. peregrina* this possibility must be investigated in future studies.

There are only scant publications on flies and maggots from Puducherry. A previous survey on filth flies in Puducherry has identified the presence of several different species- *Musca domestica, M. sorbens, Stomoxys calcitrans, Calliphora* sp.*, Fannia canicularis, Ophyra* sp.*, Sarcophaga* sp. *and Hippelates* sp (19). To our knowledge, there are no published molecular barcoding studies from Puducherry; however, there are a few reports from Karnataka in South India and some states in North India. For instance, a study undertaken to barcode fly species prevalent in poultry farms in and around Bengaluru district in Karnataka has identified five species which include *M. domestica, Chrysomya megacephala, Hydrotaea capensis, Hermetia illucens and Sarcophaga ruficornis* (9). Two other studies have been published on molecular barcoding of forensically important flies from India, one from Maharashtra and the other from North India (Himachal Pradesh, Punjab, Haryana and Uttarakhand) (20, 21).

Among the different flies identified in the current study, the presence of *Sarcophaga peregrina*, the flesh fly has been recorded throughout India. However, there are only a limited number of gene sequences of this species from India in the public data base. It has been used for PMI estimation and is also known to cause myiasis in both humans and animals (2, 22). There is limited published data on *Hemipyrrelia ligurriens* from India and only a handful of gene sequences are available in the public data base (23). This fly is also of forensic significance and myiasis caused by *H. ligurriens* has been reported in animals (11, 24). *Chrysomya megacephala*, commonly known as the oriental latrine fly is of forensic importance and is also known to cause accidental myiasis in open wounds (25, 26). There are several partial COI gene sequences of this fly species from different regions of India in the public database, but not from Puducherry.

Apart from species identification, molecular methods find special application in epidemiological investigations and evolutionary studies. Molecular epidemiological studies of flies assume significance in the context of global warming, climate and environmental changes as highlighted and predicted by a modelling study on *Chryosmya bezziana* (27). This study highlights the recent reports on expansion of the myiasis causing fly, *Chrysomya bezzian*a(Old World Screw worm Fly- OWSF)) into newer geographical regions (27). Typically, OWSF prefers tropical and subtropical climates and is widespread throughout tropical and Sub-Saharan Africa, the Middle East region, the Indian subcontinent, from Southeast Asia to China, and the Philippines to Papua New Guinea. However, recently, probably due to climate change and through the commercial movement of cattle OWSF has been expanding out of its typical range (27). Haplotype network and phylogenetic analysis are useful in exploring the genetic relatedness between strains from different regions and can be very informative in inferring the geographical origin of the strains.

Though molecular methods offer several advantages, they are not without limitations. Technical expertise and specialized instruments are necessary. It is costly as well but is being rapidly made available in routine settings.

In conclusion, we found COI based molecular method as a versatile tool for identification of maggots and this needs to be promoted in clinical and forensic settings in India. Additionally, this study generated molecular sequence data on two less studied fly species of medical importance in south India, namely *S. peregrina* and *H. ligurriens*. In future, there is a need for detailed molecular barcoding studies on flies with a large number of samples from diverse epidemiological and geographic settings which can expand our knowledge on genetic diversity in India.

## Data Availability

The datasets presented in this study can be found in online repositories. The names of the repository/repositories and accession number(s) can be found in the article/**Supplementary Material**.

## Supplementary material

Supplementary Additional File 1 Haplotype table for all three species.
